# Body composition, but not insulin resistance, influences postprandial lipemia in patients with Turner's syndrome

**DOI:** 10.20945/2359-3997000000287

**Published:** 2020-08-24

**Authors:** Rodrigo de Azeredo Siqueira, Aluana Santana Carlos, Joana Costa d'Avila, Adalgiza Mafra Moreno, Estela Luz Alves, Maria Lucia Fleiuss de Farias, Laura Maria C. Mendonça, Marilia Martins Guimarães

**Affiliations:** 1 Universidade de Nova Iguaçu Faculdade de Medicina Laboratório de Pesquisa Pré-Clínica Nova Iguaçu RJ Brasil Laboratório de Pesquisa Pré-Clínica, Faculdade de Medicina, Universidade de Nova Iguaçu, Nova Iguaçu, RJ, Brasil; 2 Universidade Federal do Rio de Janeiro Departamento de Endocrinologia Rio de Janeiro RJ Brasil Departamento de Endocrinologia, Universidade Federal do Rio de Janeiro, Rio de Janeiro, RJ, Brasil; 3 Universidade Federal do Rio de Janeiro Departamento de Reumatologia Rio de Janeiro RJ Brasil Departamento de Reumatologia, Universidade Federal do Rio de Janeiro, Rio de Janeiro, RJ, Brasil

**Keywords:** Hyperlipidemia, hormone replacement therapy, Turner's syndrome, estrogen replacement therapy, insulin resistance

## Abstract

**Objective:**

The aim of the present study was to examine the influence of body composition and insulin resistance on the magnitude of postprandial lipemia in patients with Turner's syndrome receiving oral versus transdermal estrogen replacement.

**Subjects and methods:**

Twenty-five patients with Turner's syndrome receiving oral or transdermal estrogen replacement were evaluated for body mass index, waist-to-hip and waist-to-height ratios, fasting glycemia, insulin, body composition (dual-energy X-ray absorptiometry), and postprandial lipid metabolism. For statistical analysis, we used parametric tests to compare numeric variables between the two subgroups.

**Results:**

We observed no difference in postprandial triglyceride levels between patients receiving oral versus transdermal hormone replacement therapy. The postprandial triglycerides increment correlated positively with the percentage of total fat mass (p=0.02) and android fat mass (p=0.02) in the transdermal group. In the oral estrogen group, a positive correlation was observed between the increment in postprandial triglycerides and waist-to-hip (p=0.15) and waist-to-height (p=0.009) ratios. No association was observed between the estrogen replacement route and insulin resistance evaluated by the homeostatic model assessment–insulin resistance (HOMA-IR) index (p=0.19 and p=0.65 for the oral and transdermal groups, respectively).

**Conclusion:**

We concluded that body composition and anthropometric characteristics possibly affect the extent of postprandial lipemia independently from the route of estrogen replacement.

## INTRODUCTION

Turner's syndrome is a disorder that affects females and is characterized by a complete or partial absence of one X chromosome associated with typical phenotypic features ( 1 ). Ovarian failure is a cardinal manifestation of Turner's syndrome, and more than 90% of the girls affected by this disorder require hormone replacement therapy to initiate puberty and complete growth ( 2 ). Additionally, women with Turner's syndrome have a threefold increase in mortality, due primarily to cardiovascular complications ( 3 ).

Evidence suggests that women with Turner's syndrome have a higher incidence of coronary heart disease than women in the general population ( 4 ). Indeed, patients with this syndrome have several risk factors for ischemic heart disease, including hypertension ( 5 ), insulin resistance ( 6 ), hyperlipidemia ( 7 ), and estrogen deficiency ( 8 ). However, other aspects of lipid metabolism, including postprandial plasma triglyceride (TG) levels, have not been studied in these patients. Postprandial lipemia is recognized as a key candidate in the atherosclerotic process, and increased postprandial triglyceride levels could be associated with coronary heart disease in women ( 8 , 9 ).

The choice of optimal hormone replacement therapy in children and adolescents with Turner's syndrome remains controversial ( 10 , 11 ). In the United States, oral conjugated equine estrogens are the most widely used formulation for estrogen replacement in affected girls ( 12 ). The oral and transdermal routes of estrogen administration seem to have important differences in terms of impact on plasma lipids and body composition ( 13 , 14 ). Still, no long-term studies have assessed the effect of combined hormone replacement therapy on lipid metabolism in patients with Turner's syndrome or otherwise healthy women of comparable age ( 15 ).

The present study was designed to evaluate the influence of body composition and insulin resistance on postprandial triglyceride levels in women with Turner's syndrome receiving oral versus transdermal estrogen therapy.

## SUBJECTS AND METHODS

### Subjects

The study was performed at Clementino Fraga Filho University Hospital at Federal University of Rio de Janeiro (UFRJ), Brazil. Informed consent was obtained from the participants, and all assessments performed in the study were approved by the institution's human research committee (CAAE Number 28584620.0.0000.8044). In all, 25 patients with Turner's syndrome (45,X and related karyotypes) and older than 20 years were recruited after providing informed written consent. A total of 11 patients used conjugated equine estrogen (Premarin, Wyeth Medica Ireland, Newbridge, County Kildare, Ireland) 0.625 mg/day from the 1st to the 25th day of the month plus 10 mg of medroxyprogesterone acetate (Provera, Pharmacia Corporation de Venezuela C.A., Valência, Venezuela) from the 15th to the 25th day of the month. The remaining 14 patients with Turner's syndrome used estradiol gel (Estreva Gel, Laboratoire Théramex, Monaco) 1.5 g/day and a similar medroxyprogesterone acetate posology as those in the oral estradiol group. On recruitment, all patients had been using these medications for a minimum of 1 year. Women with diabetes mellitus or preexisting renal or cardiovascular disease were excluded from the study, along with smokers and patients using aspirin or lipid-lowering agents. All included patients had consented to participate in the study after full explanation of the purpose and nature of the study procedures, in compliance with the Declaration of Helsinki.

### Study protocol

After a 12-hour overnight fast, an intravenous catheter was inserted into one of the participants' forearm vein for blood sampling. Each participant was given a meal test containing 50 g of fat per square meter of body surface area. The meal consisted of whole milk, chocolate, milk cream, coconut milk, butter, and one cracker. The composition of the meal was 61.7% of fat, 29.6% of carbohydrates, and 8.7% of protein. After the meal, the subjects were not allowed to eat for 8 hours but were given free access to water. Blood samples were drawn before the meal and every 2 hours after the meal over a 4-hour period.

Blood samples were collected in the fasting state for measurement of levels of glucose, total cholesterol, HDL cholesterol, triglycerides, and insulin. Postprandial triglyceride levels were measured in blood samples collected at baseline and after fat overload to evaluate the postmeal increment in triglyceride levels (from baseline to 2 and 4 hours).

Plasma was separated immediately after blood collection by centrifugation at 3,000 rpm and 4 °C for 10 minutes. Levels of triglycerides, total and HDL cholesterol, glucose, leukocytes, and platelets were measured by standard laboratory techniques. LDL cholesterol was calculated using the Friedewald's formula ( 16 ).

Insulin levels were determined by the chemiluminescence method using double antibodies specific to human insulin. The reference values ranged from 3 to 16 μUI/mL, the intra-assay coefficient of variation (CV) was 3.9%, and the interassay CV was 8.1%. The homeostatic model assessment for insulin resistance (HOMA-IR) index was calculated using the formula by Matthews et al., *i.e.* [insulin (in mU/mL) X glucose (in mmol/L)]/22.5, and the ratio HOMA-IR was used as a measure of insulin resistance.

### Anthropometric and body composition measurements

Body weight, height, waist, and hip circumference were measured following standardized procedures ( 17 ). Whole-body composition was evaluated using dual-energy X-ray absorptiometry (DXA; Lunar DPX-L PED, Lunar Radiation Corporation, Madison, WI, USA). DXA scanning determined the participants' whole-body and trunk fat mass (%), lean mass (g) content, and distribution of body fat (%; android or gynoid). The DXA scanning radiation dose was 0.8 mRem. A daily test was performed using a manufacturer-supplied spine phantom. All results were interpreted by the same professional. The CV between body composition measurements was ≤ 1.0%.

### Statistical analyses

The statistical analyses were performed using the statistical software SAS, version 6.04 (SAS Inc., Cary, NC, USA). To compare continuous numeric variables between the two subgroups, Student's *t* or Mann-Whitney test was used. Analysis of variance (ANOVA) for repeated measures was executed to evaluate the three moments of triglyceride level assessments in each group, and the multiple comparison Bonferroni test was applied to identify the moments that differed among each other. Spearman's correlation coefficient was used to evaluate the correlation between numerical variables. The criterion adopted for significance was the 5% level (p < 0.05).

## RESULTS

The baseline characteristics of the study participants are shown in Table 1 . No statistically significant differences were observed between groups concerning age; body mass index; total cholesterol and fractions, triglycerides, HOMA-IR index, glucose, and insulin levels; waist and hip measurements; and waist-to-hip and waist-to-height ratios. The percentages of body fat, gynoid fat, and lean mass were similar between groups. Android fat mass was greater in patients receiving transdermal estrogen replacement (p = 0.019) ( Table 2 ).

**Table 1:** Baseline clinical characteristics of the patients with Turner's syndrome in the groups of oral (n = 11) and transdermal (n = 14) estrogen replacement

	Group	Mean	SD	*P value*
Age (years)	Transdermal	24.9	5.2	0.46
	Oral	26.5	4.9	
Glucose (mg/dL)	Transdermal	78.2	11.2	0.51
	Oral	76.1	3.2	
Insulin (mU/L)	Transdermal	5.07	5.71	0.32
	Oral	5.52	3.21	
HOMA-IR	Transdermal	1.067	1.395	0.25
	Oral	1.031	0.593	
Total cholesterol (mg/dL)	Transdermal	171.3	28.6	0.12
	Oral	193.1	40.1	
HDL cholesterol (mg/dL)	Transdermal	51.6	9.9	0.066
	Oral	59.2	9.5	
LDL cholesterol (mg/dL)	Transdermal	104.2	23.2	0.64
	Oral	115.6	40.8	
Triglycerides (mg/dL)	Transdermal	76.6	27.4	0.21
	Oral	91.8	32.3	
Waist (cm)	Transdermal	80.6	11	0.072
	Oral	72.8	9.5	
Hip (cm)	Transdermal	88.2	9.1	0.074
	Oral	81.5	8.7	
Waist/hip ratio	Transdermal	0.9	0.1	0.76
	Oral	0.9	0.1	
Waist/height ratio	Transdermal	55.9	8.3	0.11
	Oral	50.8	7.3	

P values were deemed significant when < 0.05 (Student's *t* test or Mann-Whitney test). SD: standard deviation; HOMA-IR: homeostatic model assessment for insulin resistance; HDL: high-density lipoprotein cholesterol; LDL: low-density lipoprotein cholesterol.

**Table 2:** Characteristics of body composition in patients with Turner's syndrome in the groups of oral (n = 11) and transdermal (n = 14) estrogen replacement

	Group	Mean	SD	*P value*
% Body fat	Transdermal	41.8	5.4	0.26
	Oral	38.7	8.0	
% Android fat	Transdermal	46.8	7.3	0.019
	Oral	38.9	8.3	
% Gynoid fat	Transdermal	47.2	5.5	0.86
	Oral	46.8	6.9	
Lean mass (g)	Transdermal	29233	3768	0.59
	Oral	28449	3469	

P values were deemed significant when < 0.05 (Student's *t* test).

**Figure 1:**
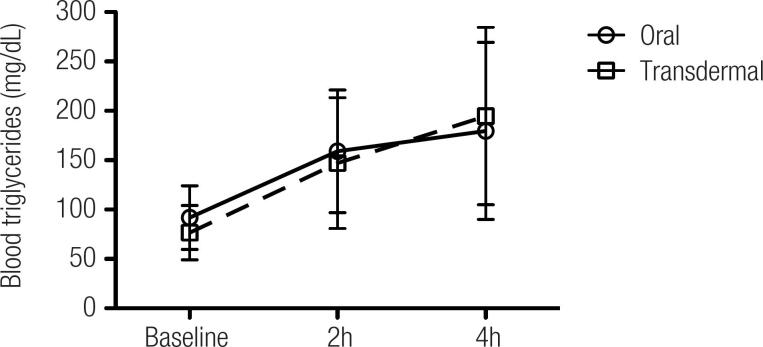
Postprandial response of triglyceride levels in patients with Turner's syndrome receiving estrogen replacement therapy. Patients receiving estrogen replacement via oral (n = 11) or transdermal (n = 14) routes had blood triglyceride levels measured before (baseline) and after (2 and 4 hours) fat overload. Statistical analysis by ANOVA for repeated measures was performed within each group (p < 0.0001) and between both groups (p = 0.17).

Levels of triglycerides after fat overload (p = 0.0001 for both subgroups) were significantly higher compared with baseline values in both subgroups of estrogen replacement. However, these increases were similar in both subgroups ( Figure 1 ). Postprandial triglyceride levels correlated positively with the percentage of fat mass (p = 0.02) and android fat mass (p = 0.002) in the transdermal group. In the oral estrogen group, the only positive correlations with postprandial triglyceride levels were observed for waist-to-hip (p = 0.15) and waist-to-height (p = 0.009) ratios ( Table 3 ). No correlations between HOMA-IR and the delta in triglyceride values were observed in both subgroups.

**Table 3:** Correlation between triglyceride levels increment (absolute and relative variations) and clinical and laboratory variables in patients with Turner's syndrome receiving oral (n = 11) or transdermal (n = 14) estrogen

		Transdermal		Oral	
		Absolute	Relative	Absolute	Relative
Age	*r_s_*	0.324	0.494	0.045	0.145
	*p*	0.26	0.07	0.89	0.67
BMI	*r_s_*	-0.026	-0.163	0.155	0.109
	*p*	0.93	0.58	0.65	0.75
Waist	*r_s_*	0.226	-0.222	0.327	0.164
	*p*	0.44	0.45	0.33	0.63
Hip	*r_s_*	0.427	0.246	0.255	0.164
	*p*	0.13	0.40	0.45	0.63
Waist/hip	*r_s_*	-0.086	**-0.574**	**0.709**	**0.618**
	*p*	0.77	**0.032**	**0.015**	**0.043**
Waist/height	*r_s_*	0.116	-0.112	**0.745**	**0.645**
	*p*	0.69	0.70	**0.009**	**0.032**
Glucose	*r_s_*	0.152	-0.187	-0.317	-0.225
	*p*	0.60	0.52	0.34	0.51
HOMA-IR	*r_s_*	0.376	-0.073	-0.155	-0.209
	*p*	0.19	0.81	0.65	0.54
% Body fat	*r_s_*	**0.609**	0.526	-0.014	0.087
	*p*	**0.02**	0.05	0.97	0.80
% Android fat	*r_s_*	**0.612**	0.251	0.200	0.218
	*p*	**0.02**	0.39	0.56	0.52
% Gynoid fat	*r_s_*	0.376	0.138	-0.318	-0.173
	*p*	0.19	0.64	0.34	0.61
Lean mass	*r_s_*	0.411	0.169	-0.491	**-0.682**
	*p*	0.14	0.56	0.13	**0.021**

P values were deemed significant when < 0.05 (Mann-Whitney test).

BMI: body mass index; HOMA-IR: homeostatic model assessment for insulin resistance.

## DISCUSSION

The present study has three main findings. First, we found no difference between transdermal versus oral route of estrogen replacement regarding postprandial triglyceride levels in patients with Turner's syndrome. Second, we observed that body composition affects postprandial triglyceride levels in both groups of patients with Turner's syndrome. Finally, we found no association between the HOMA-IR index and postprandial triglyceride levels in patients with Turner's syndrome.

The route of estrogen replacement did not affect the magnitude of postprandial lipemia. Studies have shown that postprandial hyperlipidemia may discriminate better the presence of coronary heart disease than fasting triglyceride levels ( 18 , 19 ). Disturbances in postprandial lipemia have also been observed in patients with type 2 diabetes ( 20 ) and individuals with visceral obesity or features of metabolic syndrome ( 21 ).

The baseline metabolic profile was similar across patients with Turner's syndrome regardless of the estrogen replacement route. Other studies have also shown that glucose and lipid metabolism is not affected by the route of hormone replacement therapy ( 14 , 22 - 24 ). Considering the estrogen replacement route, the transdermal group showed a higher percentage of android fat. This may be explained by the fact that replacement with oral estradiol prevents changes in total body fat and progression towards central accumulation of body fat, whereas transdermal estradiol prevents only changes in lean body mass ( 25 ).

Our study found a positive correlation between waist-to-hip ratio and postprandial triglyceride levels, as well as postprandial triglyceride levels in both groups, as seen in some studies with postmenopausal women ( 26 ), patients with polycystic ovary syndrome ( 27 ), and middle-aged healthy subjects ( 28 ). The waist-to-height ratio in the oral estrogen group was also associated with variations in triglyceride levels, and this ratio could also be a marker of triglyceride elevation in patients with Turner's syndrome. We found no studies evaluating the association of the waist-to-height ratio with postprandial lipemia.

Another important observation of our study was that total fat and android distribution of fat – which corresponds to centralized fat – correlated with the variation in triglyceride levels in the transdermal group, as described by Mekki et al. in a study with adult women ( 29 ).

Insulin resistance assessed by the HOMA-IR index was not associated with postprandial triglyceride response in our study. In contrast, many studies associate insulin resistance and postprandial lipemia in different situations ( 26 , 27 , 30 , 31 ). This can be explained by the fact that beta cell failure, rather than insulin resistance, appears to be the primary defect in glucose homeostasis in adult patients with Turner's syndrome ( 32 - 35 ). Another explanation for the lack of association between postprandial triglyceride response and insulin resistance may be the small number of participants in our study.

This study contributes important information to a field of little knowledge in Turner's syndrome. Although substantial evidence demonstrates the transdermal route to be more favorable on triglyceride levels, the findings of the present study indicate that both the transdermal and oral routes have similar effects on fasting or postprandial triglyceride levels. Our study also demonstrates that body composition can be an important modifiable factor in this context.

We conclude that anthropometric measurements and body composition, but not insulin resistance, could be important contributing factors to an exaggerated postprandial triglyceride lipoprotein response in patients with Turner's syndrome, regardless of the route used for estrogen replacement.
